# Incidence of structural vocal fold abnormalities associated with vocal fold polyps

**DOI:** 10.1016/S1808-8694(15)30596-6

**Published:** 2015-10-18

**Authors:** Claudia Alessandra Eckley, João Swensson, André de Campos Duprat, Fernanda Donati, Henrique Olival Costa

**Affiliations:** 1 PhD in Medicine - FCMSCSP, Fellow in Professional Voice - Thomas Jefferson University - Philadelphia, Assistant Professor.; 2 ENT Physician, Student at the Department of Otorhinolaryngology - Santa Casa de SP.; 3 PhD in Medicine - Otorhinolaryngology Department - Santa Casa de SP, Instructor-Professor - Otorhinolaryngology Department Santa Casa de SP.; 4 ENT Physician, Student at the Department of Otorhinolaryngology - Santa Casa de SP.; 5 PhD in Medicine - University of São Paulo, Adjunct Professor of the Otorhinolaryngology Department - SP. Otorhinolaryngology Department - Santa Casa de São Paulo

**Keywords:** larynx, vocal folds, polyp, voice

## Abstract

Phonotrauma is considered the main cause of vocal fold polyps (VFP). However, the authors believe that an underlying anatomical deviation could render the vocal folds more susceptible to such trauma.

**Aim:**

To prove this hypothesis a retrospective chart review was carried out to correlate the surgical findings of patients with VFP.

**Material and Methods:**

The charts of thirty-three patients who underwent surgery for excision of VFP were reviewed: 21 had right VFP, 10 had left VFP and 2 had bilateral lesions.

**Results:**

Associated lesions were reported in 27 patients (14 lesions on the opposite VF and 13 on the ipsilateral VF): 10 opposite nodules, 12 sulcus vocalis, 3 cysts, and 2 capillary engorgement.

**Discussion and Conclusions:**

The high incidence of associated anatomical lesions to the VF (63%) suggests that patients with these minor underlying anatomical deviations are more vulnerable to vocal abuse, probably because they present abnormal glottic closure and an irregular vibratory margin.

## INTRODUCTION

Current literature states that the main etiology of vocal fold polyps is sudden vocal trauma, which causes hematoma formation in Reinke's space that can initiate an inflammatory process, which would be associated with polyp formation^1^, ^2^, ^3^.

Vocal fold polyps are usually located in the anterior two-thirds of the vocal fold^4^, and they can eventually be found in other places (anterior and posterior commissures); they are usually bilateral, they can be pedicled or sessile, usually seen in men^1^, ^3^, ^5^, ^6^.

Speech trauma is very common in our country, however individuals respond differently to such trauma. There are those who develop polyps, others develop nodules and others do not develop injuries on the vocal folds after a speech trauma. Things like glottic configuration and exposure to chemical products and allergens have been used to explain the different types of speech-trauma lesions^1^, ^2^, ^3^, ^4^, ^5^, ^6^. More recent studies discuss the different amounts of fibronectin and hyaluronic acid on the vocal folds of men and women, and that also explains why nodules prevail in women and polyps in men^6^. Among other possible factors, we believe that there may be some prior anatomical change in the vocal folds, the so called minimum structural lesions, which may predispose certain individuals to have these speech-trauma related lesions. Since these alterations are associated with capillary ectasia, they would render these folds more predisposed to hematoma formation.

In order to contribute to the pathophysiology of vocal fold polyps, the present investigation tries to find a correlation between vocal fold polyps and the structural alterations that happen to the contralateral vocal fold or to the ipsilateral one.

## MATERIALS AND METHODS

We carried out a retrospective study, based on the charts of 33 consecutive patients submitted to laryngeal micro-surgery to remove a vocal fold polyp between July of 1997 and August of 2003, in a tertiary University Hospital, after being approved by the Research Ethics Committee of the institution (CEP 39/03). Of all the patients, 18 were men (55%) and 15 (45%) were women, with mean age of 42 years.

The polyp was clinically diagnosed through video-laryngoscopy and confirmed during surgery. The patients who had infiltrative processes or that of deposit diseases were taken off the study.

During surgery in our institution, we always look for minimum structural alterations on the vocal folds, by manipulating them with a spatula or another long blunt instrument.

By analyzing the charts, the lesions were grouped in the following fashion: the associated lesions were described as reactional lesions on the contralateral fold, epidermoid cyst, mucous cyst, minor stria sulcus, major stria sulcus, pocket-type sulcus, capillary ectasia and anterior commissure microdiaphragm, and the lesion side was described as ipsi or contralateral to the polyp.

## RESULTS

**Figure 1:**
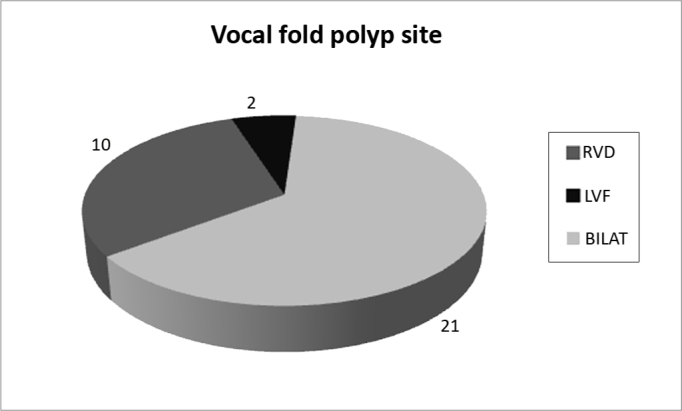
Location of polyps on the vocal folds.

Of all our patients, 21 (63.6%) had polyps on their right vocal fold (RVF), 10 (30.3%) had polyps on their left vocal fold, and 2 (6.1%) had polyps on both vocal folds (Figure 1).

**Figure 2:**
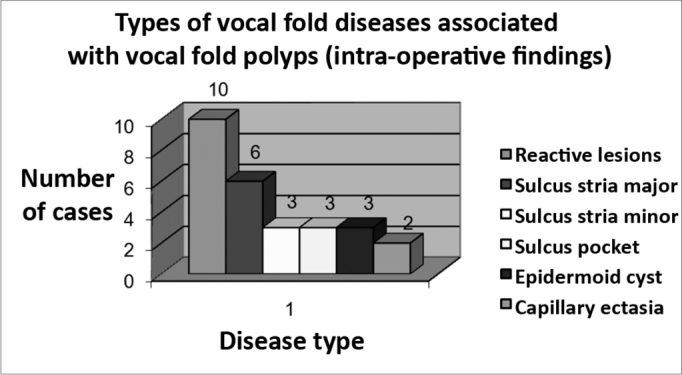
Types of vocal fold lesions associated with vocal fold polyps.

We studied 27 associated lesions on vocal folds during the surgery: 10 (37%) of them were of the nodular thickening reaction-type lesions, 6 (22.2%) were stria major sulcus, 3 (11.1%) were epidermoid-type cyst, minor stria sulcus and pocket-type sulcus, 2 (7.4%) capillary ectasia. We did not find mucous-type cysts, or microdiaphragm on the anterior commissure (Figure 2). Except for reactional lesions, we observed 51.51% (17/33) os structural lesions associated to vocal fold polyps.

Of the patients with description of stria major-type sulcus, half had polyps on their vocal folds and the other half had it on their contralateral fold.

The epidermoid-type cyst was found in one case, on the contralateral vocal fold, and in two cases on the ipsilateral vocal fold. The stria minor sulcus and the pocket-type sulcus surprisingly had the same statistics and incidence.

Capillary ectasia were found in the same vocal fold where the polyps were found.

None of the lesions were found bilaterally.

**Figure 3:**
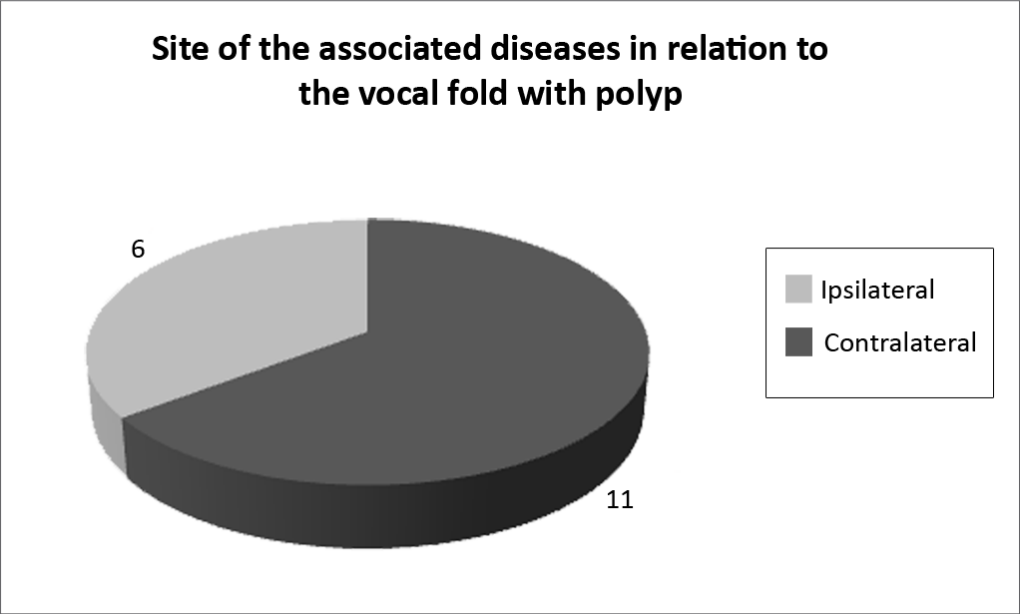
Location of associated lesions (ruling out nodular-type lesions) in relation to vocal fold with a polyp.

Of all the structural lesions found (17), 6 were contralateral and 11 were ipsilateral (Figure 3).

## DISCUSSION

Our initial hypothesis that a pre-existing lesion, because it alters vocal fold vibration dynamics, would render the patient more vulnerable to vocal trauma proved to be very much justifiable, because of the 27 lesions found, 17 were considered pre-existing lesions (cyst, sulcus, capillary ectasia). Therefore, 51% of the patients with polyps already have some pre-existing structural alteration.

This preliminary study is part of a larger one, which is still ongoing, aiming at providing a greater understanding of the factors associated with polyps on the vocal folds. We noticed structural lesions in 63% of those patients with polyps who were operated. We believe these pre-existing lesions participate in an aerodynamic change in vocal fold vibration patterns and, coupled to vocal abuse, lead to hematoma in Reinke's area, thus creating vocal fold polyps. These structural lesions change vocal fold layers that already have its own anatomical structure adapted to the patient's vibratory pattern9-10. These lesions could interfere in vocal fold coaptation, creating an irregular mucosal wave during speech production. An irregular mucosal wave could expose Reinke's area to a structural attack^9^.

The predominance of male patients in our study matches the pattern found in the literature; however, there was no overwhelming predominance of males, maybe because women go to doctors more often than men. Moreover, females, because they have more mass in their vocal folds, have a lower pitch, which impacts further their social lives, more than it would impact their male counterparts.

Surprisingly, the predominance of polyps found on the right vocal fold is twice that on the left vocal fold. This data could be associated with a cerebral dominance; however that was not the scope of this study.

The contralateral contact lesions found in 37% of the patients confirm the suspicion that the polyp's impact on the contralateral otherwise healthy vocal fold, on the long run, may change the fold's epithelial layer^6^, ^7^, ^8^, ^9^.

Among minimum structural lesions, sulcus were the more commonly found lesions (70%), being found 4 times more often than cysts (17%), and 6 times more than capillary ectasia (12%). Capillary ectasia that could predispose the patient to a higher risk of hematomas^11^ had low incidence.

The present investigation attempts to show the importance of evaluating the polyp-related lesions during surgery, thus detecting a patient's vulnerability as to disease recurrence^9^, ^10^, ^11^. It is also very important to detect the lesions in the pre-operative exams (despite diagnostic difficulties), in order to better plan the surgery with a possible approach of the associated lesions.

Although this study is still preliminary, it suggests a close relation between vocal fold polyps and the minimum structural lesions, thus better explaining the higher susceptibility of some individuals towards developing vocal fold polyps after vocal trauma.

## CONCLUSIONS

The rate of structural lesions associated with vocal fold polyps suggests that anatomical changes in the vocal fold can be associated with the pathophysiology of vocal fold polyps. These associated structural lesions must be always investigated in order to achieve a better treatment planning.
